# The Emerging Role of Phosphodiesterases in Movement Disorders

**DOI:** 10.1002/mds.28686

**Published:** 2021-06-21

**Authors:** Roberto Erro, Niccoló E. Mencacci, Kailash P. Bhatia

**Affiliations:** ^1^ Department of Medicine, Surgery and Dentistry “Scuola Medica Salernitana” University of Salerno Baronissi Italy; ^2^ Department of Neurology Northwestern University Feinberg School of Medicine Chicago IL USA; ^3^ Department of Clinical and Movement Neurosciences UCL Queen Square Institute of Neurology, National Hospital for Neurology and Neurosurgery London United Kingdom

**Keywords:** cyclic nucleotides, Huntington disease, ADCY5, adenylyl cyclases, PDE

## Abstract

Cyclic nucleotide phosphodiesterase (PDE) enzymes catalyze the hydrolysis and inactivation of the cyclic nucleotides cyclic adenosine monophosphate and cyclic guanosine monophosphate, which act as intracellular second messengers for many signal transduction pathways in the central nervous system. Several classes of PDE enzymes with specific tissue distributions and cyclic nucleotide selectivity are highly expressed in brain regions involved in cognitive and motor functions, which are known to be implicated in neurodegenerative diseases, such as Parkinson's disease and Huntington's disease. The indication that PDEs are intimately involved in the pathophysiology of different movement disorders further stems from recent discoveries that mutations in genes encoding different PDEs, including *PDE2A*, *PDE8B*, and *PDE10A*, are responsible for rare forms of monogenic parkinsonism and chorea. We here aim to provide a translational overview of the preclinical and clinical data on PDEs, the role of which is emerging in the field of movement disorders, offering a novel venue for a better understanding of their pathophysiology. Modulating cyclic nucleotide signaling, by either acting on their synthesis or on their degradation, represents a promising area for development of novel therapeutic approaches. The study of PDE mutations linked to monogenic movement disorders offers the opportunity of better understanding the role of PDEs in disease pathogenesis, a necessary step to successfully benefit the treatment of both hyperkinetic and hypokinetic movement disorders. © 2021 The Authors. *Movement Disorders* published by Wiley Periodicals LLC on behalf of International Parkinson and Movement Disorder Society

The cyclic nucleotides cyclic adenosine monophosphate (cAMP) and cyclic guanosine monophosphate (cGMP) act as intracellular second messengers for many signal transduction pathways and modulate a number of processes in the central nervous system (CNS), including neurogenesis, apoptosis, plasticity, as well as sleep, sensorimotor gating, mood stability, memory, and other cognitive functions.^1^, ^2^ cAMP and cGMP levels are tightly regulated through a fine balance between their synthesis, which is mediated by the activity of adenylyl and guanylyl cyclases (AC and GC, respectively), and their degradation.^1^, ^3^

Cyclic nucleotide phosphodiesterase (PDE) enzymes are responsible for the breakdown of cAMP and cGMP by hydrolysis of phosphodiester bonds, thereby directly regulating the intracellular levels of these second messengers. Over the years, mounting evidence has suggested that alterations in PDE expression, and consequently of cyclic nucleotides levels and their downstream targets, might occur with aging and in different age‐related diseases, including Alzheimer's disease,^1^, ^4^ and more recently their implication also has been suggested for several movement disorders, including, but not limited to, Huntington's disease (HD)^5^ and Parkinson's disease (PD).^6^ The notion that PDEs might be intimately implicated in the pathogenesis of different movement disorders has been further corroborated by the evidence that mutations in several *PDE* genes are responsible for rarer genetic conditions primarily manifesting with movement disorders.^7^, ^8^, ^9^

In this review, we aim to provide a translational overview of the preclinical and clinical data on PDEs, the role of which is emerging in the field of movement disorders, offering a novel venue for a better understanding of their pathophysiology and ultimately for the development of alternative therapeutic approaches.

## Brain Expression and Function of PDEs

Several neurotransmitters exert their actions via membrane‐bound G‐protein–coupled receptors, triggering a series of intracellular signaling cascades that are linked to the regulation of second messenger cytosolic concentrations, among which cyclic nucleotides play a pivotal role.^10^

**FIG. 1:**
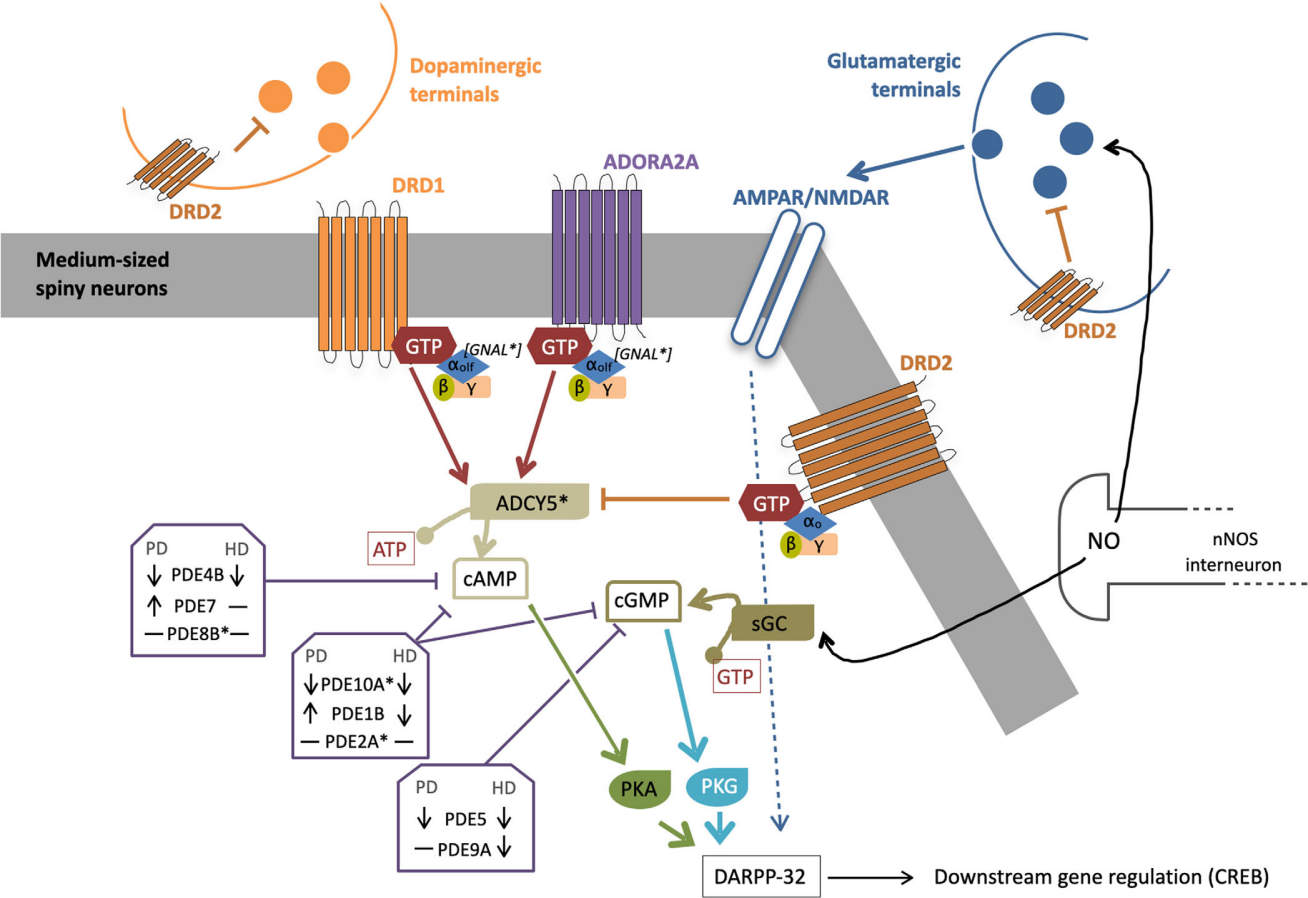
Overview of the cyclic nucleotide signaling in stratal medium‐size spiny neurons with a summary of the PDE level alteration in HD and PD. Asterisk indicates that the gene has been implicated in monogenic movement disorders. ADCY5, adenylyl cyclase 5; ADORA2A, adenosine A2a receptor; AMPAR, α‐Amino‐3‐hydroxy‐5‐methyl‐4‐isoxazolepropionic acid receptor; ATP, adenosine triphosphate; cAMP, cyclic adenosine monophosphate; cGMP, cyclic guanosine monophosphate; CREB, cAMP response element binding protein; DARPP‐32, dopamine‐ and cyclic‐AMP‐regulated phosphoprotein 32 kDa; DRD1, dopamine receptor D1; DRD2, dopamine receptor D2; GDP, guanosine diphosphate; GNAL, guanine nucleotide‐binding protein G(olf) subunit alpha; GTP, guanosine‐5′‐triphosphate; HD, Huntington's disease; NMDAR, *N*‐methyl‐d‐aspartate receptor; nNOS, neuronal nitric oxide synthase; NO, nitric oxide; PD, Parkinson's disease; PDE, phosphodiesterase; PKA, protein kinase A; PKG, protein kinase G; sGC, soluble guanylyl cyclase. [Color figure can be viewed at wileyonlinelibrary.com]

Increased cytosolic concentrations of cyclic nucleotides drive the activation of protein kinase A (PKA) and protein kinase G (PKG) and their downstream pathways.^11^, ^12^ Among these, the cAMP‐regulated phosphoprotein molecular mass 32 kDa (DARPP‐32) is thought to play a critical role in mediating many of the neuromodulatory effects of dopamine (DA) on local striatal GABAergic and glutamatergic transmission. Furthermore, it induces the transcriptional activation of cAMP response element binding protein (CREB), which has been reported to play a major role in cognitive and motor functions (Fig.1).^13^, ^14^, ^15^ PKG is also a potent activator of striatal DARPP‐32. Its phosphorylation of DARPP‐32 is rapid, transient, and requires a coincidental increase in intracellular calcium levels induced after glutamatergic stimulation of *N*‐methyl‐d‐aspartate receptor, AMPA, and metabotropic glutamate 5 receptors. Acting together, the PKA/PKG‐mediated pathways finely regulate long‐term changes in striatal synaptic efficacy.^16^ Alterations of these cascades have been demonstrated in basal ganglia disorders and are therefore likely to be critically involved in the planning and execution of complex motor behaviors. As mentioned earlier, CREB is one of the PKA/PKG target substrates that increases the levels of different neurotrophic factors, including the brain‐derived growth factor (BDNF), thus enhancing neuronal growth and development on one side, but also initiates gene transcription involved in long‐term synaptic plasticity on the other.^14^, ^15^, ^16^, ^17^, ^18^

In the striatum, cyclic nucleotides signaling is controlled by a variety of different neurotransmitters, including DA, glutamate, and adenosine. Several lines of evidence would suggest that activation of AC‐cAMP‐PKA signaling in striatal medium spiny neurons (MSNs) facilitates corticostriatal transmission, enhancing the excitatory effects of glutamatergic transmission. The excitatory responses induced by *N*‐methyl‐d‐aspartate receptor are further potentiated by D1 and reduced by D2 receptor stimulation.^10^ It is likely that stimulation of postsynaptic AC‐cAMP‐PKA signaling is primarily driven by D1‐like receptors given that they are positively coupled to AC, whereas D2 receptor activation produces the opposite effect by inhibiting AC‐cAMP‐PKA signaling at both presynaptic and postsynaptic levels (Fig. 1).^19^

In contrast, the intracellular concentration of cyclic nucleotides is further determined by the activity of PDEs, which mediate their degradation into their corresponding monophosphate nucleosides. In mammals, PDEs are encoded by 21 genes, which produce 11 distinct PDE families (PDE1–PDE11), which are classified based on their structural similarities and splice variants.^20^ PDEs can be further stratified based on their substrate specificity: PDEs 4, 7, and 8 are cAMP specific; PDEs 5, 6, and 9 are cGMP specific; whereas PDEs 1, 2, 3, 10, and 11 can hydrolyze both cyclic nucleotides (Fig. 1).^1^, ^2^, ^3^, ^20^, ^21^

Recent work has been performed to characterize PDE expression in different CNS areas. It has been shown that the PDEs most abundant in the basal ganglia are PDE1B and PDE10A, which are equally expressed in the caudate nucleus.^21^ The level of PDE1B mRNA in the caudate nucleus is 10‐ to 100‐fold higher than PDE1C and PDE1A, respectively.^21^ Similarly, PDE10A mRNA concentrations in the caudate nucleus and nucleus accumbens are at least 10‐fold higher than in any other CNS areas.^21^ Recent work has suggested that PDE2A is as highly expressed as PDE10A in the striatum,^9^ but it is also highly enriched in the hippocampus and other cortical regions. Two other PDE isoenzymes, PDE4B and PDE8B, have been implicated in the pathogenesis of movement disorders.^1^, ^5^, ^19^ PDE4B mRNA is the most highly expressed isoenzyme of the PDE4 family in the brain, with similar levels expressed in the substantia nigra, putamen, thalamus, and spinal cord.^21^ The highest PDE8B levels in brain are found in the caudate, cortex, and hippocampus.^21^

Compared with PDE7A that is highly expressed in many peripheral organs, PDE7B is dominant in the CNS, with the highest expression levels in the caudate, nucleus accumbens, and cortex as compared with relatively low levels in the cerebellum.^21^ Conversely, PDE3 mRNA levels are higher in the cerebellum than in all other brain areas, while PDE5, PDE6, PDE9, and PDE11 expressions in the CNS are low in comparison with both other PDE levels and their expression in other tissues.^21^

It is likely that different PDE isoforms act collectively in striatal neurons to modulate cyclic nucleotides signaling within specific subcellular compartments and to further regulate fine tuning of the electrical activity patterns and synaptic plasticity changes of striatal projecting neurons (for a review, see Threlfell and West^10^). Given that these abnormal firing patterns have been demonstrated in disorders of the basal ganglia, modulation of PDEs might reverse abnormal striatal neuronal excitability and synaptic plasticity. For instance, this has been demonstrated in a genetic alpha‐synuclein‐related model of PD, in which the application of the PDE5 inhibitor zaprinast fully restored aberrant long‐term depression to normal conditions via the stimulation of a cGMP‐PKG‐dependent intracellular signaling pathway.^22^

In the following sections, we present the role of PDEs in specific movement disorders. To this aim, a literature search was conducted on September 2020 using the PubMed‐NCBI database and the following search terms: (1) “phosphodiesterase*” OR “PDE” AND “Huntington” OR “Parkinson*” OR “movement disorder” OR “basal ganglia” OR “striatum”; and (2) “phosphodiesterase*” OR “PDE” AND “gene mutation.” Only articles written in English were considered, and the bibliographies of the retrieved articles and of relevant review articles were also checked. The final list of the included articles was identified according to the aim of the current work to provide an integrated and synthesized overview of the current state of knowledge.

## Huntington's Disease

Findings in both animal models and patients would suggest an impairment of cyclic nucleotide signaling in HD.^5^ Reduced cAMP levels and CREB activity have been demonstrated in both the cortex and striatum of the HD mouse model,^23^ which could be possibly owing to increased cyclic nucleotide degradation by PDEs.^24^ The most implicated PDE in HD is PDE10A given that it is particularly enriched in the striatum,^21^, ^25^ but there is also suggestion that PDE4A, PDE1B, PDE5, PDE9A, and PDE3 might be playing a role in HD pathogenesis.^26^, ^27^, ^28^ Tables 1 and 2 summarize the evidence from preclinical and clinical studies, respectively, specifically investigating PDE activity and cyclic nucleotides signaling in HD.

**TABLE 1:** Summary of the Preclinical Studies Investigating PDEs Activity and Cyclic Nucleotide Signaling in HD Models

First Author (Year of Publication)	HD Model	Main Results	Conclusions
Gines (2003)^23^	Genetic HD model (*Hdh* ^*Q111*^ knock‐in mice) and related striatal cell model	Reduced CRE signaling in *Hdh* ^*Q111*^ striatum cAMP levels in *Hdh* ^*Q111*^ striatum were decreased from an early age (10 weeks) and postmortem Reduced CRE signaling in cultured *Hdh* ^*Q111*^ striatal cells was associated with cytosolic CREB that mirrored diminished cAMP synthesis	Diminished cAMP levels amplify the early HD cascade by decreasing CRE‐regulated gene transcription and altering energy‐dependent processes
Hebb (2004)^29^	Genetic HD model (transgenic R6/2 mice carrying about 140–150 CAG repeats and R6/1 mice carrying about 115 CAG repeats integrated at a different site within the mouse genome)	PDE10A mRNA and protein levels decline in the striatum of R6/1 and R6/2 HD mice before onset of motor symptoms The rate of reduction in PDE10A mRNA and protein is more rapid in R6/2 compared with R6/1 mice Striatal PDE1B mRNA levels decline in R6/1 and R6/2 HD mice, but to a lesser extent than PDE10A PDE4A mRNA levels are relatively low in the striatum and do not differ between age‐matched WT and transgenic HD mice	Regulation of PDE10A and PDE1B, but not PDE4A, mRNA levels is dependent on the relative expression of or number of CAG repeats, leading to dysregulation of cAMP and cGMP striatal signaling
Hu (2004)^44^	Genetic HD model (transgenic R6/1 and R6/2 mice)	PDE10A2 is the predominant isoform expressed in the striatum Its mRNA levels are reduced because of a transcription initiation impairment rather than by posttranscriptional mRNA instability induced by mHTT The mouse and human PDE10A2 promoters are highly conserved	mHTT determines PDE10A2 transcriptional dysregulation affecting its promoters
DeMarch (2007)^30^	QA‐treated rats to serve as HD model	In QA rats treated with the PDE4 inhibitor rolipram, striatal lesion size was about 62% smaller than in QA rats treated with an inert vehicle Rolipram‐treated QA rats had a significant increase of the levels of activated CREB in the striatal MSNs	PDE4 inhibition exerts beneficial effects in the QA rodent model of striatal excitotoxicity
DeMarch (2008)^31^	Genetic HD model (transgenic R6/2 mice)	Rolipram‐treated R6/2 mice survived longer and showed less severe neurological dysfunction than the vehicle‐treated ones Brain volume, striatal atrophy, size and morphology of striatal neurons, prevention of neuronal intranuclear inclusions, and attenuation of microglial reaction confirmed a neuroprotective effect of rolipram Rolipram was effective in increasing significantly the levels of activated CREB and BDNF in striatal spiny neurons	PDE4 inhibitor rolipram induces neuroprotective effects possibly through increased levels of activated CREB, of which BDNF represents a target gene
Giampà (2009)^32^	Genetic HD model (transgenic R6/2 mice)	Rolipram prevented CREB sequestration into striatal neuronal intranuclear inclusions Rolipram reduced parvalbuminergic striatal interneuron degeneration as assessed by cell counts Rolipram rescued motor coordination and motor activity deficits in R6/2 mice	CREB recruitment in nuclear aggregates might drive neuronal death, whereas a minimal threshold of soluble nuclear CREB, which is promoted by PDE4 inhibition, enhances neuronal survival; this effect was demonstrated in highly vulnerable interneurons (ie, parvalbuminergic GABAergic interneurons) and paralleled motor behavior benefits in treated mice
Giampà (2009)^42^	QA‐treated rats to serve as HD model	PDE10A inhibition with the TP‐10 compound increased significantly the levels of activated CREB in the striatal spiny neurons Striatal lesion size was half, and the surviving cell number was several times higher in mice treated with TP‐10 than in the vehicle‐treated group	PDE10A inhibitor TP‐10 exerts a neuroprotective effect in an excitotoxic model of HD
Giampà (2010)^41^	Genetic HD model (transgenic R6/2 mice)	PDE10A inhibition with the TP‐10 compound increased striatal and cortical levels of phosphorylated CREB and BDNF, and reduced striatal and cortical cell loss, the formation of striatal neuronal intranuclear inclusions, and the degree of microglial activation PDE10A inhibition improved motor deficits of symptomatic R6/2 mice	Targeting cAMP and CREB signaling through PDE10A inhibition improves HD striatal pathology and motor deficits
Puerta (2010)^54^	3‐NP‐treated rodents to serve as HD model	The PDE5 inhibitor sildenafil improved motor abnormalities, reduced the loss of striatal DARPP‐32 protein levels and lesion volumes, and decreased calpain activation produced by 3‐NP Striatal phosphorylated CREB levels, along with the expression of BDNF, were significantly increased in sildenafil‐treated rats	PDE5 inhibitors protect against 3‐NP‐induced striatal degeneration by reducing calpain activation and by promoting survival pathways
Giralt (2013)^39^	Genetic HD model (transgenic R6/1 mice)	PDE10A inhibition with papaverine increased cAMP, but not cGMP, levels and activated cAMP‐dependent protein kinase (PKA) signaling in hippocampal neurons Papaverine treatment improved spatial and object recognition memories in R6/1 mice	PDE10A inhibition improves cognition in R6/1 mice by activating cAMP‐dependent signaling in nonstriatal (ie, hippocampal) neurons
Leuti (2013)^46^	Genetic HD model (transgenic R6/2 mice)	PDE10A levels were higher in the spiny neurons, as well as in parvalbuminergic, somatostatinergic, and calretininergic interneurons, of R6/2 mice compared with WT mice, whereas its level was lower in the striatal cholinergic interneurons In the TP‐10 (PDE10A inhibitor)‐treated R6/2 mice, PDE10A levels were lower than in the control group in the MSNs, whereas they were higher in all subsets of striatal interneurons except for the cholinergic ones	PDE10A is increased in the spiny neurons of R6/2 mice striatum and, by hydrolyzing greater amounts of cyclic nucleotides, is likely to contribute to cell damage in HD; the beneficial effect of TP‐10 in HD models might be explained by the increase of the availability of cyclic nucleotides
Beaumont (2016)^38^	Genetic HD model (Q175 knock‐in mice and R6/2 mice)	Despite the observed loss of PDE10A, PDE10 inhibition with the PF‐02545920 compound drives striatal cAMP and cGMP elevation, both of which are required to boost diminished corticostriatal input and indirect pathway output in symptomatic HD models PDE10A inhibition starting at presymptomatic ages mitigates the emergence of mHTT‐induced corticostriatal transmission deficits and aberrant striatal projecting neurons excitability	PDE10A loss may represent a homeostatic adaptation to maintain impaired cyclic nucleotide signaling in HD; nonetheless, PDE10A inhibitors are effective in raising striatal cAMP and cGMP and, consequently, reducing or preventing the emergence of HD neurophysiological deficits in symptomatic and presymptomatic HD models, respectively
Harada (2017)^40^	Genetic HD model (transgenic R6/2 mice)	PDE10A inhibition with TAK‐063 suppressed the reduction of striatal BDNF and prevented striatal atrophy PDE10A inhibition reduced seizure frequency and development of motor deficits, but not of motor coordination impairment, and improved deficits in procedural learning	PDE10A inhibition reduces striatal neurodegeneration and ameliorates some behavioral deficits in R6/2 mice
Tanaka (2017)^24^	Genetic HD model (transgenic R6/2 mice carrying about 130 CAG repeats) Autopsied brains from 6 patients with HD and 3 control subjects	HTT forms a ternary protein complex with the scaffolding protein DISC1 and PDE4 Pathological cross‐seeding between DISC1 and mHTT aggregates was observed in both human and R6/2 brains In R6/2 mice, reductions in soluble DISC1 led to aberrant increase of PDE4 activity Exogenous expression of a modified DISC1, which binds to PDE4, but not mutant HTT, normalized PDE4 activity and ameliorated anhedonia in the R6/2 mice	Cross‐seeding of mutant HTT and DISC1 and the resultant changes in PDE4 activity may underlie the pathology of a specific subset of mental manifestations of HD
El‐Abhar (2018)^28^	3‐NP‐treated rodents to serve as HD model	The PDE3 inhibitor cilostazol exerted anti‐inflammatory effects at the striata level documented by the pronounced reduction of the toll‐like receptor 4 protein expression and the inflammatory cytokine IL‐6 Cilostazol reduced IL‐6 downstream signal, promoting the level of SOCS3, abating the phosphorylation of JAK‐2 and STAT‐3, and inducing phosphorylation of the protein kinase B/glycogen synthase kinase‐3β/CREB complex	Contrary to the 3‐NP effects, PDE3 inhibition could preserve striatal dopaminergic neurons and improve motor coordination through anti‐inflammatory and antiapoptotic effects
Chakroborty (2020)^27^	Genetic HD model (BACHD transgenic line 5 [TG5])	Compared with WT controls, MSNs exhibited decreased spike probability during cortical stimulation in TG5 animals Large increases in onset latency of cortically evoked spikes and decreases in spike probability were observed in fast‐spiking interneurons recorded in TG5 animals Acute systemic administration of the PDE9A inhibitor PF‐04447943 significantly decreased the onset latency of cortically evoked spikes in MSNs recorded in WT and TG5 rats PDE9A inhibition also increased the proportion of MSNs responding to cortical stimulation and reversed deficits in spike probability observed in TG5 rats	Facilitation of striatal cGMP signaling and glutamatergic corticostriatal transmission induced by PDE9A inhibition could restore striatal function

PDE, phosphodiesterase; HD, Huntington's disease; CRE, cAMP response element; cAMP, cyclic adenosine monophosphate; CREB, CRE binding protein; WT, wild type; cGMP, cyclic guanosine monophosphate; mHTT, mutant huntingtin; QA, quinolinic acid; MSN, middle‐size spiny neuron; BDNF, brain‐derived neurotrophic factor; 3‐NP, 3‐nitropropionic acid; DARPP‐32, dopamine‐ and cAMP‐regulated phosphoprotein 32 kDa; PKA, protein kinase A; SOCS3, suppressor of cytokine signalling 3; JAK‐2, Janus Kinase 2; STAT‐3, signal transducers and activators of transcription 3; TP‐10, 2‐{4‐[‐pyridin‐4‐yl‐1‐(2,2,2‐trifluoro‐ethyl)‐1H‐pyrazol‐3‐yl]‐phenoxymethyl}‐quinoline succinic acid.

**TABLE 2:** Summary of the Clinical Studies Investigating PDEs Involvement in HD

First Author (Year of Publication)	Methods	Main Results	Conclusions
Russell (2014)^47^	PET with the PDE10A ligand [^18^F]MNI‐659 in 8 symptomatic and 3 presymptomatic subjects with HD	Lower striatal [^18^F]MNI‐659 uptake in HD than in healthy control subjects, with presymptomatic subjects showing intermediate basal ganglia binding potentials; striatal [^18^F]MNI‐659 uptake correlated with UHDRS Motor subscale, the molecular marker (ie, an estimation of the genetic burden adjusted for age), and striatal atrophy	[^18^F]MNI‐659 is potentially capable of assessing the extent of disease in early HD
Niccolini (2015)^50^	Multimodal MRI and PET with the PDE10A ligand [^11^C]IMA107 in 12 early presymptomatic HD subjects with an estimated 90% probability of 25 years before the predicted clinical onset	PDE10A availability was decreased in striatum and pallidum and increased in motor thalamic nuclei in subjects with HD as compared with healthy control subjects Connectivity‐based analysis revealed PDE10A decreases confined in the sensorimotor‐striatum and in striatonigral and striatopallidal projecting segments The ratio between PDE10A expression in motor thalamic nuclei and in striatopallidal projecting striatum was the strongest correlate of higher probability of symptomatic conversion	PDE10A imaging demonstrates very early neurochemical changes in premanifest HD gene carriers and can be used to track disease progression
Russell (2016)^48^	PET with the PDE10A ligand [^18^F]MNI‐659 in a longitudinal cohort of 6 symptomatic and 3 presymptomatic subjects with HD; PET imaging sessions were performed approximately 1 year apart	In the HD cohort, the mean annualized rates of decline in [^18^F]MNI‐659 uptake in the caudate, putamen, and globes pallidus were 16.6%, 6.9%, and 5.8%, whereas the annualized reduction in signal in striatal regions was less than 1% in healthy control subjects	[^18^F]MNI‐659 PET imaging of PDE10 might serve as a useful biomarker to track HD progression
Wilson (2016)^51^	Volumetric MRI and PET with the PDE10A ligand [^11^C]IMA107 in 12 early presymptomatic HD subjects	PDE10A availability was reduced in HD subjects within the insular cortex and occipital fusiform gyrus compared with healthy control subjects, whereas no atrophy was detected	Dysregulation of PDE10A signaling could be an early phenomenon in extrastriatal brain areas that are relevant for the regulation of cognitive and limbic functions that are impaired in HD
Fazio (2020)^49^	PET with the PDE10A ligand [^18^F]MNI‐659 in 45 subjects with HD, of whom 35 participated in the longitudinal phase and were scanned again at 18 months from baseline; subjects further underwent PET imaging of D2/3 receptors and structural MRI	Striatal and pallidal PDE10A availability was lower in HD subjects than in healthy control subjects Striatal and pallidal [^18^F]MNI‐659 uptake progressively declined in the premanifest stages but plateaued between stages 1 and 2 The percentage decline of [^18^F]MNI‐659 uptake was greater than that of D2/3 receptor availability and of volumetric changes	[18 F]MNI‐659 PET imaging might track HD progression more sensitively than the dopamine receptor and volumetric imaging methods

PDE, phosphodiesterase; HD, Huntington's disease; PET, positron emission tomography; [^18^F]MNI‐659, [^18^F]MNI‐659 (2‐(2‐(3‐(4‐(2‐[18F]fluoroethoxy)phenyl)‐7‐methyl‐4‐oxo‐3,4‐dihydroquinazolin‐2‐yl)ethyl)‐4‐isopropoxyisoindoline‐1,3‐dione); UHDRS, Unified Huntington's Disease Rating Scale; MRI, magnetic resonance imaging.

The striatal mRNA expression of PDE4A in R6/2 mice (ie, one of the first transgenic HD models created by inserting a copy of the five untranslated regions, exon 1 with about 150 CAG repeats, and part of intron 1 of the human HD gene into the mouse genome) has been found to be proportionately decreased as the animals age.^29^ However, significant decrease in striatal CREB activity has also been demonstrated in R6/2 mice.^29^ Therefore, it has been suggested that reduced expression of PDE4 could be compensatory and would depend on the effect of mutant huntingtin (mHTT) protein that sequesters DISC1, a protein that normally binds to and inhibits PDE4B, which in turn may lead to reduced cAMP levels and PKA and CREB activity.^24^ Of note, the PDE4 inhibitor rolipram increases CREB phosphorylation exerting neuroprotective effects in both the quinolinic acid (QA) rat model^30^ and genetic R6/2 mouse model of HD.^31^ As mentioned earlier, PDE4 is mainly expressed in the cortex and would drive the psychiatric, but not motor, phenotype of HD,^24^ although rolipram has been further found to rescue motor coordination and activity deficits of R6/2 mice.^32^

Compensatory changes caused by direct effects of mHTT onto cycling nucleotide signaling have been further postulated for PDE1B and PDE10A. PDE1B mRNA levels have been reported to be reduced in R6/2,^33^ R6/1,^34^ and N171‐82Q HD transgenic mouse models,^35^ as well as cDNA obtained from mRNA of symptomatic patients with HD,^36^ suggesting a mHTT‐dependent decrease in PDE1B expression. The PDE1 inhibitor, vinpocetine, has been reported to ameliorate both biochemical and behavioral abnormalities in toxic models of HD.^37^

A number of studies have shown that PDE10A inhibition is able to rescue behavioral, neurodegenerative, and electrophysiological deficits in HD animal models, which correlates with an increase of CREB activity and BDNF levels in both the striatum and the cortex.^38^, ^39^, ^40^, ^41^, ^42^ However, conflicting with these beneficial results, PDE10A mRNA expression has been shown to be consistently reduced in both cell^23^ and animal^29^ models of HD, as well as in brain tissue or cerebrospinal fluid of patients with HD.^23^, ^43^, ^44^, ^45^ Similarly to PDE4A and PDE1B, it has been speculated that this reduction may be compensatory to a primary decrease in cAMP levels driven directly by mHTT.^41^ An alternative possible answer was suggested by a study investigating PDE10A levels in a specific subpopulation of striatal neurons. PED10A levels were found to be increased in MSNs and other subsets of striatal interneurons (ie, parvalbuminergic, somatostatinergic, and calretininergic interneurons) in R6/2 transgenic HD mice compared with the wild‐type mice, whereas striatal cholinergic interneurons PDE10A levels were lower.^46^ Importantly, densitometric studies of the whole striatum showed lower PDE10A immunoreactivity in the R6/2 than in the wild‐type mice.^46^ This suggests that, although PDE10A levels are lower in the striatum in toto, it might be overexpressed in MSNs, where it inhibits cyclic nucleotides and downstream signaling. In contrast, low levels of PDE10A found in cholinergic interneurons might be possibly related to their selective resistance to HD neurodegeneration.^5^ It should be noted, however, that the observed increase of PED10A levels in MSNs occurred at 4 and 9 weeks of age in R6/2 mice, whereas a significant decrease was observed at 13 weeks of age,^46^ which might further suggest that PDE10A levels might initially increase in a compensatory fashion and subsequently decrease throughout disease progression.

In humans, a positron emission tomography (PET) study has shown that striatal binding of the PED10A ligand, [^18^F]MNI‐659, was significantly lower in both patients with HD and premanifesting HD gene carriers than in healthy control subjects, and strongly correlated with brain atrophy, disease severity, and the burden of pathology (ie, an estimation of the genetic burden adjusted for age).^47^ Longitudinal PET studies have further shown that the mean annualized rate of decline in [^18^F]MNI‐659 caudate binding was about 16% compared with less than 1% in healthy volunteers, suggesting it might efficiently track HD progression and in a more sensitive way than with both functional and volumetric imaging methods that are currently used.^48^, ^49^ Another study using the PED10A ligand, [11]C‐IMA107, showed bidirectional changes in PDE10A expression in premanifest HD gene carriers, with lower binding in the striatum and pallidum and increased binding in motor thalamic nuclei than in healthy volunteers.^50^ The ratio between higher PDE10A expression in motor thalamic nuclei and lower PDE10A expression in striatopallidal projecting striatum was found to be the strongest predictor of symptomatic conversion.^50^ Notably, these changes were observed early before symptomatic onset and when no brain atrophy was detectable,^50^ indicating that reduction of PDE10A expression does not merely reflect loss of MSNs. Decreased PED10A expression could be further observed in extrastriatal areas, which could account for the development of cognitive and psychiatric symptoms in HD.^51^ Taken together, this body of work suggests that PDE10A levels in patients with HD are prominently and progressively reduced through disease progression and might be primarily linked to the phenotypic manifestations, which is also supported, as discussed in more detail later, by the identification of loss‐of‐function *PED10A* mutations causing chorea in humans.^52^ This would contradict the preclinical evidence suggesting that PED10A alteration might be merely compensatory to cAMP/cGMP signaling changes driven by mHTT, as well as the arguably weak evidence of neuroprotective effects of PDE10A inhibitors. In fact, in humans, the Pfizer compound PF‐02545920 (ie, PDE10A inhibitor) is the only one that has currently completed a 6‐month multicenter phase 2 study (AMARYLLIS) investigating two doses (5 and 20 mg) versus placebo and failed to meet its primary end point (ie, the total motor score of the Unified Huntington's Disease Rating Scale) as publicly announced by Pfizer in a press release.^53^

Finally, the PDE5 inhibitor sildenafil has been found to reduce striatal projection neurons loss, increase CREB activity and BDNF levels, as well as improve the phenotype of the 3‐nitroproprionic neurotoxic rat model of HD,^54^ which further points to a significant role of cGMP signaling in HD pathogenesis, as also suggested by a recent study that used the selective PDE9A inhibitor PF‐04447943 in transgenic HD rats and demonstrated a normalization of cortically evoked firing of striatal MSNs.^27^ Because PDE9A is a cGMP‐specific enzyme, its inhibitors facilitate striatal cGMP signaling and, in turn, corticostriatal transmission. Similarly to earlier, however, it remains to be clarified whether the observed alterations are primarily linked to HD pathogenesis or reflect compensatory changes.

### Parkinson's Disease

There are somewhat conflicting results as to whether PDEs are implicated in the pathogenesis of PD (Tables 3 and 4 summarize the results of the preclinical and clinical studies investigating PDEs and cyclic nucleotides involvement in PD, respectively). An in vitro study showed that decreased BDNF expression determines loss of dopaminergic neurons in the substantia nigra,^55^ suggesting that cyclic nucleotide signaling might be altered in PD.^56^ In fact, there is evidence of a reduction of cGMP levels in the striatum and globus pallidus, along with an increase of striatal cAMP levels in 6‐hydroxydopamine (6‐OHDA) models.^56^ Such alteration might depend on an increase of PDE1B^57^, ^58^ and a decrease of PDE10A expression,^57^, ^59^ respectively. This type of bimodal regulation, which has also been demonstrated in animal models unrelated to PD,^60^, ^61^ would be because of the fact that PDE1B is highly expressed in the striatonigral pathway, whereas PDE10A would be more expressed in the striatopallidal pathway.^62^ A reduction of PDE10A activity also has been demonstrated *in vivo* in patients with PD: decreased striatal and pallidal binding of ^11^C‐IMA107 (ie, a highly selective PDE10A radioligand) was found in both patients with early and patients with more advanced PD, which correlated with the duration of the disease, as well as the severity of both motor symptoms and motor complications.^63^, ^64^ However, these results have not been confirmed^65^ and quite counterintuitively, inhibition of PDE10A^66^, ^67^ exerts anti‐inflammatory and neuroprotective effects in ‐MPTP models of PD.

**TABLE 3:** Summary of the Preclinical Studies Investigating PDEs Involvement and Cyclic Nucleotides Activity in PD

First Author (Year of Publication)	Model	Main Results	Conclusions
Chalimoniuk (2004)^69^	MPTP mouse model	MPTP induced an increase of GC activity in the striatum, which in turn caused a marked enhancement of cGMP formation No change in PDE activity has been detected	cGMP signaling pathway may contribute to striatal dopaminergic degeneration in PD, independently of PDE activity
Sancesario (2004)^58^	6‐OHDA rat model	In the lesioned striatum basal cGMP levels were reduced and cAMP levels were increased, but cGMP‐PDE and cAMP‐PDE activities were both increased in basal and Ca^2+^‐calmodulin‐stimulated conditions PDE1B was overexpressed in striatal neurons	Dopamine deafferentation induces downregulation of the NO‐cGMP pathway in the striatum along with an upregulation of PDE1B‐dependent cyclic nucleotide metabolism
Chalimoniuk (2007)^68^	MPTP mouse model	MPTP (and high dosage of l‐dopa in normal mice) induced the NOS/GC/cGMP pathway This effect was significantly lower and cGMP‐dependent PDE activities were elevated in normal mice administrated with low dose of l‐dopa	cGMP levels were significantly less elevated in mice treated with lower doses of l‐dopa as compared with those treated with higher doses, although a similar activation of GC was observed, suggesting a differential mechanism of PDE activity in the two conditions
Giorgi (2008)^85^	6‐OHDA rat model	Unilateral lesion of substantia nigra led to an increase in cAMP but a decrease in cGMP levels in the ipsilateral basal ganglia In dyskinetic animals, chronic l‐dopa treatment led to an absolute decrease in cAMP and cGMP levels Zaprinast (PDE5 inhibitor) reduced the severity of LID and partly prevented the decrease in cyclic nucleotides	A significant reduction in cyclic nucleotides levels is observed at the peak of LID, which might result from increased catabolism through PDE overactivity
Yang (2008)^73^	MPTP mouse model	Rolipram (ie, PDE4 inhibitor) significantly attenuated MPTP‐induced dopamine depletion in the striatum and reduced the loss of TH‐positive neurons in the substantia nigra The effects were not due to alteration in the pharmacokinetics or metabolism of MPTP	PDE4 inhibition could have a therapeutic potential in PD
Morales‐Garcia (2011)^80^	Primary cultured neurons (SH‐SY5Y), primary rat mesencephalic cells, and LPS‐rat model	S14 (ie, PDE7 inhibitor) reduced microglial activation and protected dopaminergic neurons S14 rescued motor impairment in LPS‐treated rats Blocking cAMP signaling via PKA inhibition reverted the neuroprotective effects of S14	PDE7 inhibition could have therapeutic potential in PD
Picconi (2011)^86^	6‐OHDA rat model	LID was associated with the loss of LTD expression at glutamatergic striatal synapses onto spiny neurons Zaprinast and UK‐343664 (both PDE5 inhibitors) were able to rescue the induction of this form of synaptic plasticity via a mechanism requiring the modulation of intracellular cGMP levels This effect on synaptic plasticity was paralleled by a significant reduction of LID	Physiological synaptic plasticity in the striatum, the abnormality of which appears to be associated with LID, can be restored by PDE5 inhibitors
Tozzi (2012)^22^	A53T‐*SNCA* transgenic mice	In old transgenic mice, impaired striatal LTD could not be restored by acute/subchronic dopaminergic treatment, whereas application of zaprinast (PDE5 inhibitor) fully restored LTD to normal conditions via stimulation of the PKG intracellular signaling pathway	Transduction pathway other than the dopaminergic one and cyclic nucleotides signaling are implicated in PD pathogenesis
Sancesario (2014)^57^	6‐OHDA rat model	Dyskinetic animals had lower levels of cAMP/cGMP during the increasing phase of dyskinesias than eukinetic animals, but their levels increased in the extinction phase of dyskinesias Both dyskinesias and the abnormal lowering of striatal cAMP/cGMP could be prevented by amantadine Total cGMP‐specific PDE activity was increased in dyskinetic animals After l‐dopa, PDE1B and PDE10A expression did not increase and, accordingly, PDE1B and PDE10A inhibitors failed to prevent dyskinesias	cAMP/cGMP signaling is involved in the development of LID, but the striatal PDE isoforms contributing to this abnormality are to be determined
Morales‐Garcia (2015)^81^	6‐OHDA rat model and primary rat mesencephalic cells	S14 or BRL50481 (two PDE7 inhibitors) increased the level of CREB S14 compound manifested mitogenic properties and elicited differentiation of stem cells toward a dopaminergic phenotype S14 induced a significant increase in the SNpc neurons in rats lesioned with 6‐OHDA	PDE7 inhibition could represent a method of replacing neurons lost in the SNpc of PD
Sharma (2015)^70^	MPTP mouse model	MPTP reduced cyclic nucleotides and dopamine levels and caused elevation in oxidative‐nitrosative stress markers The PDE1 inhibitor vinpocetine enhanced cyclic nucleotide levels, attenuated oxidative‐nitrosative stress, and restored dopamine level in MPTP‐treated animals	PDE1 inhibition could have a therapeutic potential in PD
Beck (2018)^88^	MPTP‐treated macaques	MR1916 (PDE10A inhibitor) consistently reduced LID in acute tests of l‐dopa optimal and suboptimal doses MR1916 did not affect the antiparkinsonian action of l‐dopa, and its effects were sustained with chronic administration of l‐dopa	Regulation of striatal cyclic nucleotides by PDE10A inhibition could be a useful therapeutic approach for LID
Hedya (2018)^84^	Rotenone‐induced rat model	Cilostazol (PDE3 inhibitor) upregulated Nurr1 expression resulting in successful preservation of the dopaminergic neurons and in marked improvement of motor performance Cilostazol revealed anti‐inflammatory activity and promoted autophagy	PDE3 inhibition could have a potential therapeutic role in PD
Russo (2018)^75^	LRRK2^G2019S^ knock‐in mice and transfected microglial cells	LRRK2 kinase activity was found to be a negative regulator of PKA activation state in microglia by affecting PDE4 activity and modulating cAMP content LRRK2 G2019S pathological mutation downregulated PKA activation and increased inflammation	PDE4 would be a putative LRRK2 effector in microglia; LRRK2 G2019S mutation may favor microglia activation, which could contribute to the progression of the pathology in LRRK2‐related PD
Lee (2019)^66^	MPTP mouse model	Papaverine (PDE10 inhibitor) reduced MPTP‐induced dopaminergic neuronal cell death and recovered locomotor activity by reducing mRNA levels of proinflammatory cytokines and enhancing the mRNA level of the anti‐inflammatory cytokine IL‐10 H89, a PKA inhibitor, reversed the anti‐inflammatory and behavioral effects of papaverine	PDE10 inhibitors exert anti‐inflammatory and neuroprotective effects via the PKA pathway
Zhong (2019)^74^	Primary cultured dopaminergic neurons (SH‐SY5Y)	FCPR16 (PDE4 inhibitor) triggered autophagy and attenuated production of reactive oxygen species and the decline of mitochondrial membrane potential in MPP^+^‐treated cells FCPR16 activated AMP‐activated protein kinase, which may initiate the autophagosome formation and promote lysosome biogenesis	PDE4 inhibition could have therapeutic potential in PD
Arakawa (2020)^87^	6‐OHDA rat model	MR1916 (PDE10A inhibitor) reduced LID without affecting the antiparkinsonian effects induced by l‐dopa	Regulation of striatal cyclic nucleotides by PDE10A inhibition could be a useful therapeutic approach for LID
Kim (2020)^67^	MPTP mouse model	MP‐10 (PF‐2545920; ie, a PDE10A inhibitor) decreased dopaminergic cell death and microglial activation MP‐10 rescued behavioral deficits in MPTP mice	PDE10 inhibition has neuroprotective and anti‐inflammatory effects in PD mouse models
Morales‐Garcia (2020)^82^	Primary cultured neurons (SH‐SY5Y), primary rat mesencephalic cells, LPS‐rat model, and 6‐OHDA rat model	PDE7 is upregulated after an injury both in the human dopaminergic cell line SH‐SY5Y and in primary rat mesencephalic cultures, as well as after LPS or 6‐OHDA injection in the SNpc of adult mice PDE7 levels are higher in degenerating dopaminergic cells and in microglia LPS and 6‐OHDA induced the expression of a PDE7 promoter	PDE7 is involved in the pathways leading to neurodegeneration and inflammatory‐mediated damage in PD

PDE, phosphodiesterase; PD, Parkinson's disease; MPTP, 1‐methyl‐4‐phenyl‐1,2,3,6‐tetrahydropyridine; GC, guanylyl cyclase; cGMP, cyclic guanosine monophosphate; cAMP, cyclic AMP; 6‐OHDA, 6‐hydroxydopamine; NO, nitric oxide; l‐dopa, levodopa; NOS, nitric oxide synthase; CREB, cAMP response element‐binding protein; LID, levodopa‐induced dyskinesia; *SNCA*, α‐synuclein; TH, tyrosine hydroxylase; PKA, protein kinase A; LPS, lipopolysaccharide; LTD, long‐term depression; PKG, protein kinase G; SNpc, substantia nigra pars compacta; Nurr1, Nuclear receptor related 1; LRRK2, Leucine‐rich repeat kinase 2; MPP^+^, 1‐methyl‐4‐phenylpyridinium; AMP, adenosine monophosphate.

**TABLE 4:** Summary of the Clinical Studies Investigating PDE Involvement in PD

First Author (Year of Publication)	Methods	Main Results	Conclusions
Casacchia (1983)^77^	Double‐blinded, placebo‐controlled, crossover trial of rolipram (ie, PDE4 inhibitor) as symptomatic add‐on treatment in 10 patients with PD	Rolipram at the dosage of 3 mg/day had no positive or negative effects compared with placebo	PDE4 inhibitor rolipram is not able to potentiate D1 stimulation
Niccolini (2015)^63^	PET with the PDE10A ligand [^11^C]IMA107 in 24 patients with moderate to advanced PD	Lower caudate, putamen, and globus pallidus [^11^C]IMA107 uptake in patients than in control subjects, which correlated with disease duration and motor severity	There is loss of striatal and pallidal of PDE10A expression that associates with PD duration and severity
Koole (2017)^65^	Volumetric MRI PET with the PDE10A ligand [^18^F]JNJ42259152 in 9 patients with early PD and 5 patients with probable PSP	In PD, striatal BP_ND_ values tended to be lower than in healthy control subjects, but this did not reach statistical significance, even after omission of partial volume correction; BP_ND_ values did not correlate with clinical features of PD	PDE10A availability was not reduced in PD, maybe because of the small sample size or short disease duration
Niccolini (2017)^78^	[^11^C]rolipram PET in 12 patients with PD with no cognitive impairment	Patients with PD showed reductions in [^11^C]rolipram VT compared with healthy control subjects, in the caudate, thalamus (prefrontal and temporal thalamic nuclei, while motor nuclei were less affected), hypothalamus, and cortex (especially posterior dorsolateral frontal cortex, medial frontal cortex, and supplementary motor area) Worse performance in spatial working memory correlated with lower [^11^C]rolipram VT values in posterior dorsolateral frontal cortex, medial frontal cortex, supplementary motor area, precentral gyrus, caudate, and prefrontal thalamic nuclei	There is loss of PDE4 expression in the striato‐thalamo‐cortical circuit in PD, which is associated with deficits of spatial working memory
Pagano (2019)^64^	PET with the PDE10A ligand [^11^C]IMA107 and with the dopamine transporter ligand [^11^C]PE2I in 56 patients with de novo to advanced PD	Loss of [^11^C]IMA107 uptake was detectable from the earliest stage of disease in both caudate and putamen, declined annually by 3.6% and 2.8%, respectively, and was associated with the gradual and progressive increase of motor symptoms	PDE10A imaging shows similar potential with dopamine transporter imaging to follow disease progression
Wilson (2020)^79^	[^11^C]rolipram PET and multimodal MRI in 12 patients with PD with and without EDS	Patients with PD with EDS showed significantly increased [^11^C]rolipram VT in the caudate, hypothalamus, hippocampus, and limbic striatum than patients without EDS Higher ESS scores correlated with increased [^11^C]rolipram VT in the caudate, hypothalamus, hippocampus, and limbic subdivisions of the striatum	Presence and severity of EDS in PD is associated with elevated PDE4 expression

PDE, phosphodiesterase; PD, Parkinson's disease; PET, positron emission tomography; MRI, magnetic resonance imaging; PSP, progressive supranuclear palsy; BP_ND_, nondisplaceable binding potential; VT, volume of distribution; EDS, excessive daytime sleepiness; ESS, Epworth Sleepiness Scale.

Notably, however, differently from 6‐OHDA models, an increase in both cAMP and cGMP activity has been found in some,^68^, ^69^ but not all,^70^ studies using 1‐methyl‐4‐phenyl‐1,2,3,6‐tetrahydropyridine (MPTP) models of PD. In fact, one study showed that motor deficits, as well as reduced cAMP/cGMP levels, could be rescued by the PDE1 inhibitor vinpocetine.^70^ Notably, some classic antiparkinsonian medications, such as selegiline and amantadine, play an inhibitory effect on PDE1A2,^71^, ^72^ which suggests a possible additional mechanism for their symptomatic effect in PD. Moreover, different preclinical studies have shown the potential therapeutic role of different PDE4 inhibitors in PD: thus, MPTP neurotoxicity can be partially reversed by restoring DA levels in mesencephalic neurons, by attenuating neuroinflammation, and by triggering autophagy.^73^, ^74^ Notably, leucine‐rich repeat kinase 2 recently has been found to modulate PKA activity by affecting PDE4 activity in microglia, modulating cAMP degradation, content, and its dependent signaling.^75^ Interestingly, PDE4 inhibitors have been shown to improve motor function in a mouse model of Batten disease, a lysosomal storage disorder, which features parkinsonism,^76^ but failed to improve parkinsonism in humans in a small, double‐blind, crossover trial.^77^ Recent PET studies using [^11^C]rolipram did demonstrate loss of PDE4 expression in several brain areas, including the striato‐thalamo‐cortical circuit in patients with PD, that, however, was not correlated with the presence and severity of motor symptoms, but with working memory deficits^78^ and excessive daytime sleepiness.^79^

The potential of PDE inhibitors in preventing dopaminergic neuronal loss has been further tested in LPS‐induced dopaminergic toxicity in rats. Hence S‐14 and BRL 50481, both of which are PDE7 inhibitors, showed similar neuroprotective effects, reducing microglial activation and neuronal loss, which were paralleled by an elevation in cAMP levels.^80^, ^81^ These results would be supported by a recent study using different in vitro and in vivo models of PD that showed a significant PDE7 upregulation taking place mainly in degenerating dopaminergic neurons and in microglia cells.^82^

Recent evidence also implicates PDE3 in PD: in a rotenone rat model, cilostazol (ie, a PDE3 inhibitor) exerted a potential protective effect by enhancing Nurr1 (a transcription factor that regulates expression of the gene encoding tyrosine hydroxylase and development of midbrain DA‐producing neurons, the mutations of which cause a rare form of adult‐onset PD^83^) and by modulating apoptosis and autophagy.^84^

Preclinical studies have also implicated abnormal cAMP/cGMP signaling in the development of levodopa (l‐dopa)‐induced dyskinesia (LID) in PD, suggesting a role of PDEs in the development of such motor complications. Thus, dyskinetic animals show lower striatal cAMP/cGMP levels at 60 minutes after l‐dopa administration compared with nondyskinetic animals, which upregulates during the extinction phase of dyskinesia (90–150 minutes after l‐dopa administration).^57^ This is in line with the evidence that striatopallidal cAMP was significantly reduced at peak of dyskinesias compared with nondyskinetic animals.^85^ Importantly, administration of the PDE inhibitor zaprinast (nonselective inhibitor of PDEs 5, 6, 9, 10, and 11) effectively reduced LID severity, preventing cyclic nucleotides level decrement.^85^ Moreover, UK‐343664 (ie, a PDE5 inhibitor) and MR1916 (ie, a PDE10A inhibitor) have also been reported to attenuate LID in 6‐OHDA rats^86^, ^87^ and parkinsonian monkeys,^88^ suggesting that PDE could, in fact, be targeted for preventing or treating LID in PD.

### Tardive Dyskinesias

In animals chronically exposed to either haloperidol or clozapine, increased PDE1B, PDE4, and PDE10A levels have been documented in both membrane and cytosolic fractions,^89^ suggesting that there is an upregulation of PDE activity contributing to abnormal cyclic nucleotide signaling. In line with this, the PDE4 inhibitor rolipram has been reported to suppress involuntary orofacial movements in haloperidol‐induced tardive dyskinesia (TD) in rats.^90^ However, the mechanisms whereby the improvement occurs are not entirely understood because, as mentioned earlier, PDE4 is mainly expressed at the cortex, which is consistent with the antipsychotic effects of PDE4 inhibitors. PDE4B genetic variants were, in fact, not associated with TD incidence and severity in patients with schizophrenia chronically treated with antipsychotics.^91^ More work is therefore needed to fully understand the role of PDEs in the genesis and as a potential therapeutic target in TD.

### Movement Disorders Resulting From *PDE* Gene Mutations

In 2010, a heterozygous mutation of *PDE8B* was first described in a German family as causative of autosomal dominant striatal degeneration (ADSD), a rare genetic disorder that is characterized by slowly progressive dysarthria, brisk deep tendon reflexes, and mild parkinsonism without tremor and poor response to l‐dopa treatment.^7^ ADSD, with onset in the fourth to fifth decade of life, is further characterized by a distinctive magnetic resonance imaging (MRI) pattern featuring symmetrical hyperintensities of the caudate, putamen, and nucleus accumbens.^7^ A few other patients with the same phenotype and imaging findings have been reported.^92^, ^93^, ^94^ In all cases, heterozygous frameshift mutations in *PDE8B* resulted in a stop codon leading to a severely truncated and dysfunctional protein.^7^, ^92^, ^93^, ^94^ Because PDE8B is highly expressed in the striatum and regulates cAMP levels, which is the second messenger of DA receptors, and these patients do not respond to l‐dopa therapy, it was proposed that ADSD is caused by a defect in DA signaling downstream of the DA receptors.

Heterozygous *PDE10A* mutations have been recently implicated in a childhood‐onset nonprogressive choreic syndrome with bilateral striatal hyperintensity on MRI.^8^, ^95^, ^96^, ^97^ All identified missense mutations (p.F300L, p.F334L/C) have been demonstrated to recurrently affect amino acid residues that are completely conserved down to invertebrate species and are located in the GAF‐B domain, likely altering the morphology of the cAMP binding pocket.^8^ Interestingly, in vitro studies showed that these mutations do not substantially impair basal PDE10A activity but severely affect the positive regulatory mechanism of cAMP binding to the GAF‐B domain on PDE catalytic activity.^8^ It was therefore proposed that dominant *PDE10A* mutations may have a strong impact on the regulation of MSNs activity, especially when MSNs are activated by high levels of cAMP.^8^ However, recent evidence showed that heterozygous mutations in the GAF‐B domain may also drive misprocessing and misfolding of the protein.^98^ Notably, another report demonstrated that loss‐of‐function biallelic *PDE10A* mutations, located in the GAF‐A domain and resulting in severe striatal PDE10A loss, are responsible for an infantile‐onset hyperkinetic movement disorder, indicating that loss of PDE10A activity is sufficient to cause chorea in humans.^52^ Interestingly, patients with biallelic *PDE10A* mutations did not show the striking abnormalities of the striata observed on imaging of patients with dominant variants in the GAF‐B domain, indicating that dominant and recessive mutations have different pathogenic mechanisms.

This work also showed that the knock‐in mouse model carrying the homologous variant identified in humans had decreased striatal PDE10A and an impaired capacity to degrade cAMP, further highlighting the critical role of PDE10A in motor control across species.^52^ However, surprisingly, these mice showed a clear hypokinetic movement disorder, suggesting that the same reduction in PDE10A activity results in radically opposite effects on movement production in humans and mice.

Finally, Salpietro et al^9^ have demonstrated *PDE2A* homozygous mutations in a single case with childhood‐onset chorea with epilepsy and cognitive impairment. In vitro functional studies showed that both cAMP and cGMP hydrolysis were decreased approximately 4‐fold and 6.5‐fold, respectively, for the mutant compared with the wild‐type PDE2A.^9^ In addition, it was reported that neither cAMP nor cGMP could increase enzyme activity, indicating a severe disruption of catalytic activity of mutated PDE2A.^9^ The high expression of PDE2A in extrastriatal brain regions^9^, ^21^ might account for the presence of additional neurological signs, beyond chorea. Recently, *PDE2A* homozygous mutations have been confirmed to cause a complex infantile syndrome consisting of paroxysmal dyskinesia with cognitive disability and electroencephalographic abnormalities or overt epilepsy.^99^

### Unsolved Questions and Conclusions

There is mounting evidence of abnormal cyclic nucleotide signaling in the development of movement disorders. Although we focused in this review on the role of PDEs, additional evidence supporting the critical role of cyclic nucleotide signaling in basal ganglia circuitry regulation and movement disorder pathogenesis stems from the discovery that mutations in *GNAL* and *ADCY5* are also a cause of hyperkinetic movement disorders, featuring dystonia, chorea, and myoclonus in variable combinations.^100^, ^101^, ^102^, ^103^, ^104^ Interestingly, patients with *ADCY5* mutations also share microstructural basal ganglia pathology with cases with *PDE10A* mutations.^105^

*GNAL* and *ADCY5* code, respectively, for Gα_olf_ and AC5, two of the main molecules involved in cAMP signaling downstream of activation of DA and adenosine receptors in MSNs of the direct and indirect pathways, respectively.^106^ Pathogenic *GNAL* mutations are loss of function, hence resulting in reduced cAMP synthesis in MSNs,^101^ while the majority of pathogenic *ADCY5* mutations cause an increase of AC5 activity, and hence an increase of cAMP production.^104^, ^107^ Importantly, the fact that mutations with opposite effect on cAMP production in MSNs result in somewhat similar movement disorders clearly indicates the extreme sensitivity of this pathway to perturbations in either direction, highlighting some of the potential pitfalls and difficulties for developing novel therapeutic strategies focused on MSNs cAMP metabolism. Nonetheless, given the relative ease of modulating cyclic nucleotide signaling, by either acting on their synthesis (ie, on AC)^107^ or on their degradation (ie, on PDEs), these could be promising therapeutic targets, despite the negative results obtained so far.

More research is, however, needed to understand whether some of the observed alterations reflect compensatory rather than primary changes. For instance, it would seem that PDE10A levels are actually reduced in humans with HD, and an approach opposite to what has been pursued in the AMARYLLIS study would consist in enhancing rather than inhibiting its activity. However, a full understanding of the delicate balance between PDE10A activity in the direct and indirect pathways, in both physiological and pathological conditions, as well as how to target PDEs in both a brain region‐specific and a compartment‐specific manner, is required.

Finally, there is a limited number of studies investigating PDE isoform expression and function in humans. The conflicting results arising from preclinical studies using different models might be further owing to the specificity of the models under investigation, which might not exactly recapitulate the pathophysiology occurring in humans. For instance, the observation that the same loss‐of‐function *PDE10A* mutation causes a hyperkinetic movement disorder in humans and a hypokinetic motor phenotype in mouse models clearly suggests that there may be species‐specific differences in the way PDE10A activity modulates the output of striatal circuits.

As far as PD is concerned, there is increasing evidence implicating cyclic nucleotide signaling in its pathogenesis, but there still remain a number of unsolved questions. If, on the one hand, PDE inhibition has been shown *in vitro* to exert neuroprotective effects, on the other, it is surprising that many studies targeted PDE isoforms, like PDE5, that are not among the most expressed in the striatum. Moreover, more work is needed to understand which specific PDE isoforms are implicated in PD pathogenesis and how each of them interacts with the underlying neurodegenerative process. For instance, when the activity of direct pathway striatal MSNs is reduced by D1 antagonism, PDE10A inhibitors increase the indirect pathway drive, therefore acting as D2‐like antagonists.^108^ Conversely, they exert D1‐like agonism, when the activity of the indirect pathway is reduced by the administration of the D2‐antagonist haloperidol.^89^ PDE10A inhibitors can hence activate both striatonigral and striatopallidal MSNs, depending on their underlying state,^109^ despite having a stronger impact on the latter.^18^ Conversely, PDE1B has a preferential modulatory effect on the striatonigral MSNs.^19^ Given that striatonigral and striatopallidal MSNs exert opposing effects on motor control, it is likely that these two PDEs interact to produce the appropriate motor output, but how this occurs remains unknown.

Therefore, more work is warranted to obtain a better understanding of the link between PDE functioning and the pathogenetic mechanisms in humans of each single condition examined in this review..

## Author Roles

1. Conception and design of the study, or acquisition of data, or analysis and interpretation of data;

2. Drafting the article or revising it critically for important intellectual content;

3. Final approval of the version to be submitted.

R.E.: 1, 2, 3

N.E.M.: 2, 3

K.P.B.: 1, 2, 3

## Financial Disclosures

R.E. receives royalties from publication of Case Studies in Movement Disorders—Common and Uncommon Presentations (Cambridge University Press, 2017). N.E.M. is funded by a Parkinson's Foundation grant. K.P.B. has received grant support from Welcome/MRC, NIHR, Parkinson's UK, and EU Horizon 2020. He receives royalties from publication of the *Oxford Specialist Handbook Parkinson's Disease and Other Movement Disorders* (Oxford University Press, 2008), *Marsden's Book of Movement Disorders* (Oxford University Press, 2012), and *Case Studies in Movement Disorders—Common and Uncommon Presentations* (Cambridge University Press, 2017). He has received honoraria/personal compensation for participating as consultant/scientific board member from Ipsen, Allergan, and Merz and honoraria for speaking at meetings and from Allergan, Ipsen, Merz, Sun Pharma, Teva, UCB Pharmaceuticals, the American Academy of Neurology, and the International Parkinson's Disease and Movement Disorders Society.

## Data Availability

No data are available.
